# Environmental effects and their impact on yield in adjacent experimental plots of high-stem and short-stem wheat varieties

**DOI:** 10.1186/s12870-024-04967-z

**Published:** 2024-04-16

**Authors:** Xiujuan Ren, Xinhua Li, Xingqi Ou, Zijuan Wang

**Affiliations:** 1 Henan Institute of Science and Technology, Xinxiang, Henan Province 453003 PR China; 2 Xinxiang Nongle Seed Industry Co., Ltd, Xinxiang, Henan Province 453002 PR China

**Keywords:** Regional wheat trials, Short-stem wheat variety, High-stem wheat variety, Marginal effects(Marginal utility), Economic yield

## Abstract

**Background:**

In regional wheat trials, when short-stem wheat varieties and high-stem wheat varieties are planted adjacent to each other in small plots, changes in their marginal plot environment can lead to bias in yield evaluation. Currently, there is no relevant research revealing the degree of their mutual influence.

**Results:**

In a regional wheat experiment, when high-stem wheat varieties and short-stem wheat varieties were planted adjacent to one another, there was no significant change in soil temperature or humidity in the high-stem wheat variety experimental plot from November to May compared to the control plot, while the soil humidity in the short-stem wheat variety experimental plot was greater than that in the control plot. In May, the soil temperature of the short-stem wheat varieties in the experimental plot was lower than that in the control plot. Illumination of the wheat canopy in the high-stem wheat variety experimental plot had a significant positive effect in April and May, while illumination of the wheat canopy in the short-stem wheat variety experimental plot had a negative effect. The chlorophyll fluorescence parameters of flag leaves in the high-stem wheat variety experimental plots showed an overall increasing trend, while the chlorophyll fluorescence parameters of flag leaves in the experimental plots of short-stem wheat varieties showed a decreasing trend. The analysis of the economic yield, biological yield, and yield factors in each experimental plot revealed that the marginal effects of the economic yield and 1000-grain weight were particularly significant and manifested as positive effects in the high-stem wheat variety experimental plot and as negative effects in the short-stem wheat variety experimental plot. The economic yield of the high-stem wheat variety experimental plot was significantly greater than that of the control plot, the economic yield of the short-stem wheat variety experimental plot was significantly lower than that of the control plot, and the economic yield of the high-stem experimental plot was significantly greater than that of the short-stem experimental plot. When the yield of the control plot of the high-stem wheat varieties was compared to that of the control plot of the short-stem wheat varieties, the yield of the control plot of the short-stem wheat varieties was significantly greater than that of the control plot of the high-stem wheat varieties.

**Conclusions:**

Based on these findings, it is concluded that plots with high-stem and short-stem wheat varieties are adjacent in regional wheat trials, the plots of high-stem wheat varieties are subject to marginal positive effects, resulting in a significant increase in economic yield; the plots of short-stem wheat varieties are subject to marginal negative effects, resulting in a decrease in economic yield. This study reveals the mutual influence mechanism of environment and yield with adjacent planting of high-stem and short-stem wheat varieties in regional wheat trials, providing a useful reference and guidance for optimizing the layout of regional wheat trials.

## Background

In the process of wheat production in China transitioning from medium yield to high yield, the middle-stem or high-stem wheat varieties that are mainly used in production will experience severe lodging and yield reduction under high yield conditions; the water and fertilizer tolerance, lodging resistance, and large spike ability of short-stem wheat varieties have attracted the attention of wheat breeders; in 1995, Chinese wheat breeders recognized the important significance of short-stem wheat variety breeding for the transition of wheat to high-yield production [1]. The plant height of the main cultivated wheat varieties in China decreased from 121.4 cm to 81.3 cm from 1950 to 2000 [2]. The plant height of newly approved wheat varieties in Hubei, Shandong, Qinghai, and Henan provinces in China from 2001 to 2020 ranged from 71.60 to 102.60 cm, and the plant height of newly approved wheat varieties has shown a decreasing trend [3–6]. The regulations for regional wheat trials in China stipulate that wheat trial plots be randomly arranged, with a plot area of 13.33 m^2^ and a 40 cm walkway between plots. Economic yields were calculated by the harvest of the whole plot [7]. However, short-stem wheat varieties have not received special attention.

Marginal effects can be divided into positive and negative effects in terms of their properties. The positive effects show that the effect zone (transitional zone, junction zone, edge zone) has better characteristics than adjacent ecosystems, such as increased productivity and species diversity. Conversely, negative effects are referred to as negative effects [8, 9]. Research by Zhang Yuping et al. revealed that, compared with that of the middle row, the yield of several high-yielding rice varieties increased by 93.8% in the edge row [10]. Similarly, Xu Yanrong et al. reported that the yield, number of grains per spike, and 100-grain weight of corn per plant gradually decreased from row 1 to row 5 in a community experiment [11]. Similarly, in a plot-planting prosomillet, the marginal maximum yield increase rate reached 207.7% with increasing ridge width [12]. Similarly, in a regional wheat experiment, the economic yield of the side row accounted for 7.41–27.08% of the actual economic yield of the plot, with an average of 14.95% [13]. Moreover, research has shown that the average edge advantage of economic yield in regional wheat trials is 30.24% [14]. In crop production, if the height of adjacent crops is different, more significant marginal effects will be produced. An intercropping experiment with corn and short kidney beans showed that the yield of the corn edge row and secondary edge row increased by 58.7% and 40.8%, respectively, compared to that of the middle row, while the yield of the short kidney bean side row and secondary side row decreased by 49.7% and 45.6%, respectively, compared to that of the middle row [15]. The surrounding conditions, such as the environmental factors of agricultural plots, are important and influence crop output and total productivity [16, 17]. Crop physiology and development can be greatly affected by numerous factors, including toxin levels, soil quality, fertility, irrigation techniques, and ecosystem variety [18, 19]. In agroforestry, crop yields near trees are significantly reduced [20]. The above research indicates the universality of marginal effects under different crops and experimental conditions.

For the purpose of creating sustainable agricultural practices and optimizing yield potential under various growing conditions, it is crucial to comprehend how differences in ecosystem dynamics, irrigation techniques, and soil characteristics affect crop performance [21]. Henan is the main planting area for wheat in China [22, 23]. The Wheat Genetic Improvement Research Center of the Henan Institute of Science and Technology has undertaken the experimental task of a regional wheat trial in the southern Huang-Huai-Hai region of China. During the arrangement of regional trials and yield measurements, it was found that when short-stem wheat varieties are planted adjacent to wheat varieties with different plant heights, their yield performance significantly changes. What is the marginal effect and impact on yield of short-stem and high-stem wheat varieties when they are planted adjacent to each other in experimental plots? This is an urgent practical problem that needs to be solved. In this study, we examined how these environmental variables affect the yield of nearby experimental plots planted with both short-stem and high-stem wheat varieties. Field experiments were conducted from 2019 to 2020. This study provides a valuable reference for yield evaluation in regional wheat trials and may provide information for future breeding and experimental design strategies.

## Experimental materials and methods

### Test varieties and their basic agronomic traits

The tested wheat varieties were Xinhuamai818 and Bainong307, and some basic agronomic traits of the two wheat varieties are shown in Table 1. Both of these wheat varieties were provided by the Wheat Genetic Improvement Research Center of Henan Institute of Science and Technology. The height of the Xinhuamai818 plants was 15.08 cm greater than that of the Bainong307 plants, and the former had a less compact plant type and certain lodging resistance. Bainong307 had a compact plant type and an average plant height of 67.2 cm in the field. It is one of the short-stem wheat varieties promoted in the Huang-Huai-Hai wheat region in recent years.

**Table 1:** Basic agronomic characteristics of these two wheat varieties

Wheat Variety	Plant Type	Plant Height (cm)	Number of Spikes Per Unit Area (10^4^/666.7m^2^)	Grain Number Per Spike	1000- Grain Weight (g)
Xinhuamai818	Less Compact Plant Type	82.36	45.05	28.02	38.89
Bainong307	Compact Plant Type	67.02	40.02	36.28	39.28

## Experimental methods

**Fig. 1:**
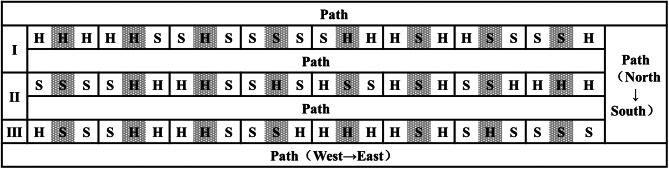
Planting map of the experimental plot (the gray area represents the research plot)

The experiment was conducted at the wheat breeding base in Zhong-Xiao-Ying Village, Hui-Xian County, Henan Institute of Science and Technology. The soil was calcareous brown soil, and the whole experimental field received consistent fertilizer and water management measures. The experimental plots were arranged in an east‒west direction, with 6 rows in each plot in a north‒south direction. The plots were 1.33 m wide and 4 m long, with a row spacing of 23 cm and a ridge width of 40 cm. The path width between the plots was 100 cm, and the seeding rate was 200,000/666.7 m^2^. The code for one experimental plot of the Xinhuamai818 wheat variety was H, and the code for one experimental plot of the Bainong307 wheat variety was S. Three experimental plots were arranged consecutively in the field as one experimental treatment or control. The three adjacent experimental plots of the Xinhuamai818 wheat variety (HHH) were used as controls for the high-stem wheat varieties; HHS, SHH, and SHS were used as the experimental treatments; SSS was the control for the short-stem wheat varieties; and SSH, HSS, and HSH were the experimental treatments for the short-stem wheat varieties. The field planting map is shown in Fig. 1. The gray plot represents the research plot; there were 3 replicates for both the experimental treatment and the control. The middle plot of the control or treatment plots was used as the research plot, in which the changes in the wheat canopy illumination, soil temperature, soil moisture content, and yield in the different plots were measured.

**Fig. 2:**
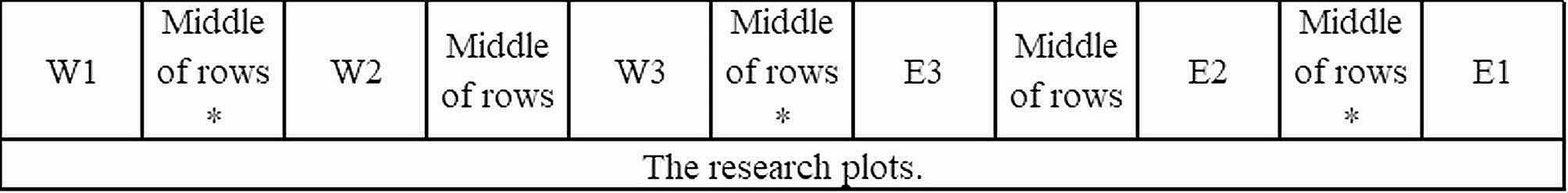
Position of climate equipment in plots (the gray plots in Fig. 1). Notes: * is the location of the climate transmitter equipment. W1, W2, and W3 are the first, second, and third rows, respectively, from west to east in the plot, and E1, E2, and E3 are the first, second, and third rows, respectively, from east to west in the plot

On October 13, 2019, after wheat planting, farmland climate monitoring equipment (from Xinyang Qihang Information Technology Co., Ltd.) was installed at the edge or in the middle row of the control and experimental plots. MS-10 soil temperature and humidity sensors were installed at a depth of 20 cm underground, and these sensors monitored and recorded soil temperature and humidity at various points in real time. An SSL10 illuminance transmitter was installed above ground, and the photosensitive detector of the transmitter was located in the upper canopy of the wheat plants. The height of the photosensitive detector was adjusted according to the dynamic growth of the wheat plants. After complete heading of the wheat plants, the height of the photosensitive detector was no longer adjusted. The farmland monitoring system collected data every 2 min and automatically uploaded the data to a computer for storage. The installation position is shown in Fig. 2.

During the early stage of wheat filling, a Handy PEA Plant Efficiency Analyzer (Hansatech Instrument Ltd., UK) was used to measure the chlorophyll fluorescence parameters of each row of wheat flag leaves. First, the flag leaves were subjected to 30 min of dark adaptation, and each treatment was repeated 6 times. The specific measurement process was performed according to the methods of Zheng Huifang [24]. The calculated chlorophyll fluorescence parameters included *φ*Po (maximum photochemical efficiency), *PIabs* (performance index based on absorbed light energy), *ABS/RC* (effective number of photochemical reaction centers), *DIO/RC* (energy dissipated per unit reaction center), *TRO/RC* (energy captured per unit reaction center for reducing QA), and *ETo/RC* (energy captured per unit reaction center for electron transfer).

### Data analysis

The average monthly values of soil temperature, soil moisture, and canopy light intensity recorded by the field monitoring system from 9 to 11 am and from 15 to 17 pm were obtained and compared from November 2019 to May 2020 for statistical analysis. The data organization and charts were completed using WPS, and the statistical analysis was completed using SPSS 18.0. The formula for calculating the marginal utility of small plots is as follows [25]: E is the marginal utility, A1 is the corresponding experimental data for the treatment, and A2 is the corresponding experimental data for the control.

$${\text{E}}(\% )\,=\,\frac{\text{(A1-A2)}\, \times \,100}{\text{A}2}$$

## Results and analysis

### Changes in the upper wheat canopy illumination, soil temperature, and soil moisture in the plots

**Fig. 3:**
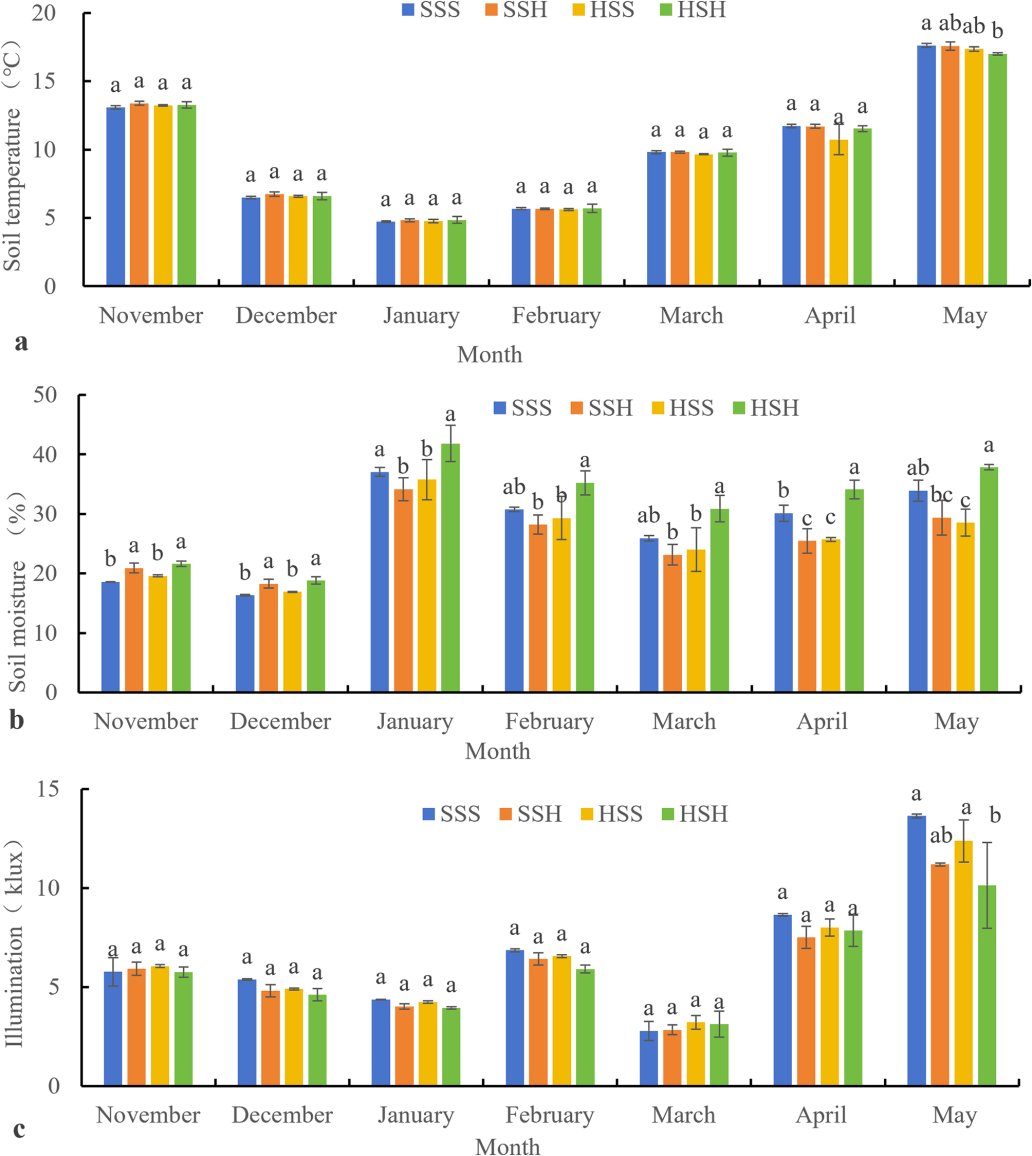
Changes in the environment of plots that have been planted with short-stem wheat varieties from November to May (**a**: changes in soil temperature, **b**: changes in soil moisture, **c**: changes in illumination of the upper canopy). Note: Different letters represent significant differences at the 0.05 level; the same applies below

As shown in Fig. 3(a), there was no significant difference in soil temperature between the treatments from November to April when the short-stem wheat varieties were planted adjacent to the high-stem wheat varieties. In May, the soil temperature in the SSS treatment was significantly greater than that in the HSH treatment and greater than that in the SSH and HSS treatments.

Figure 3(b) shows that the soil moisture in the HSH treatment was greatest from November to May and was greater than that in the other treatments. The soil moisture in the HSH treatment in November and December was significantly greater than that in the SSS and HSS treatments, but there was no significant difference in the soil moisture between the HSH and SSH treatments. From January to May, the soil moisture in the HSH treatment was significantly greater than that in the SSH and HSS treatments. Except for April, the soil moisture in HSH was greater than that in SSS, but the difference was not significant.

Figure 3(c) shows that there was no significant difference in canopy illumination among the treatments from November to March. In April, the illumination of the SSH, HSS, and HSH is lower than that of the SSS but not significantly lower. In May, the HSH had the lowest illumination, which was significantly lower than that of the SSS, and the illumination of the SSH and HSS was lower than that of the SSS but not significantly lower.

**Fig. 4:**
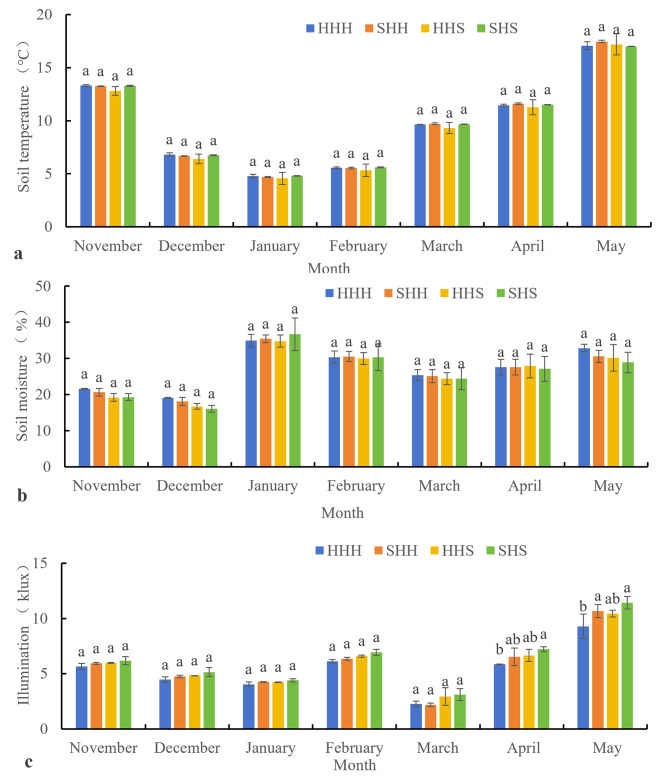
Changes in the environment of plots that have been planted with high-stem wheat varieties from November to May (**a**: changes in soil temperature, **b**: changes in soil moisture, **c**: changes in illumination of the upper canopy)

As shown in Fig. 4(a and b), there was no significant difference in soil moisture or soil temperature between HHH and the other treatments from November to May. Figure 4(c) shows that from November to March, there was no significant difference in canopy illumination between the treatments and the control group. In April and May, the canopy illumination in the SHS treatment was significantly greater than that in the HHH treatment. SHH and HHS had greater canopy illumination than did HHH, but the difference was not significant.

### Changes in the chlorophyll fluorescence parameters of flag leaves during the early stage of grouting in the experimental plots

**Fig. 5:**
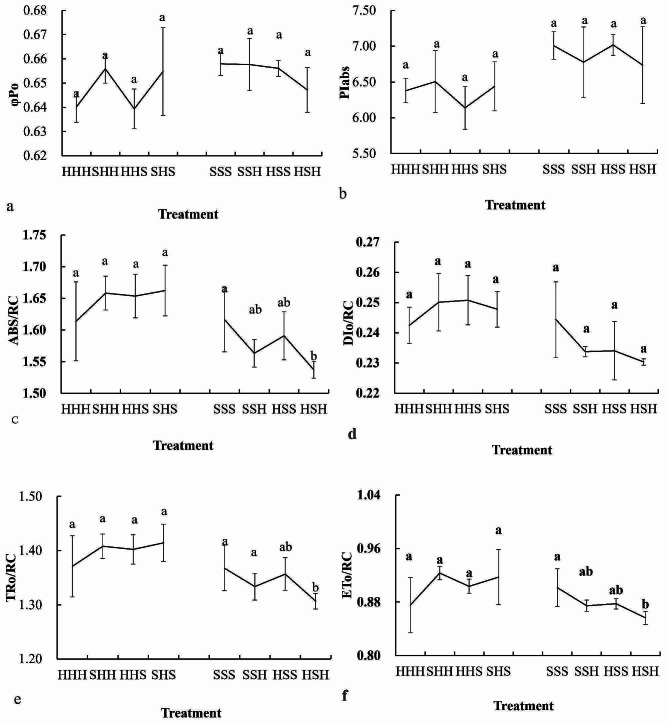
Changes in the chlorophyll fluorescence parameters of flag leaves during the early stage of grouting in the experimental plots. (**a**: changes in *φPo*, **b**: changes in *PIabs*, **c**: changes in *ABS/RC*, **d**: changes in *DIO/R*C, **e**:changes in *TRO/RC*, **f**:changes in *ETo/RC*)

As shown in Fig. 5(a, b, c, d, e, f), the chlorophyll fluorescence parameters, such as *φPo*, *PIabs*, *ABS/RC*, *DIO/RC*, *TRO/RC*, and *ETo/RC*, of the SHS flag leaves were greater than those of the HHH flag leaves, but the difference was not significant. Compared with that in the HHH plots, the chlorophyll fluorescence in the flag leaves of the high-stem wheat varieties in the experimental plots was generally greater.

The chlorophyll fluorescence parameters, such as *φPo*, *PIabs*, *ABS/RC*, *DIO/RC*, *TRO/RC*, and *ETo/RC*, of the HSH flag leaves were lower than those of SSS, and *ABS/RC*, *TRO/RC*, and *ETo/RC* were significantly lower than those of SSS. Compared with that in the SSS plot, the chlorophyll fluorescence in the flag leaves in the short-stem wheat variety experimental plots was generally lower.

### Changes in plot production and three yield factors

**Fig. 6:**
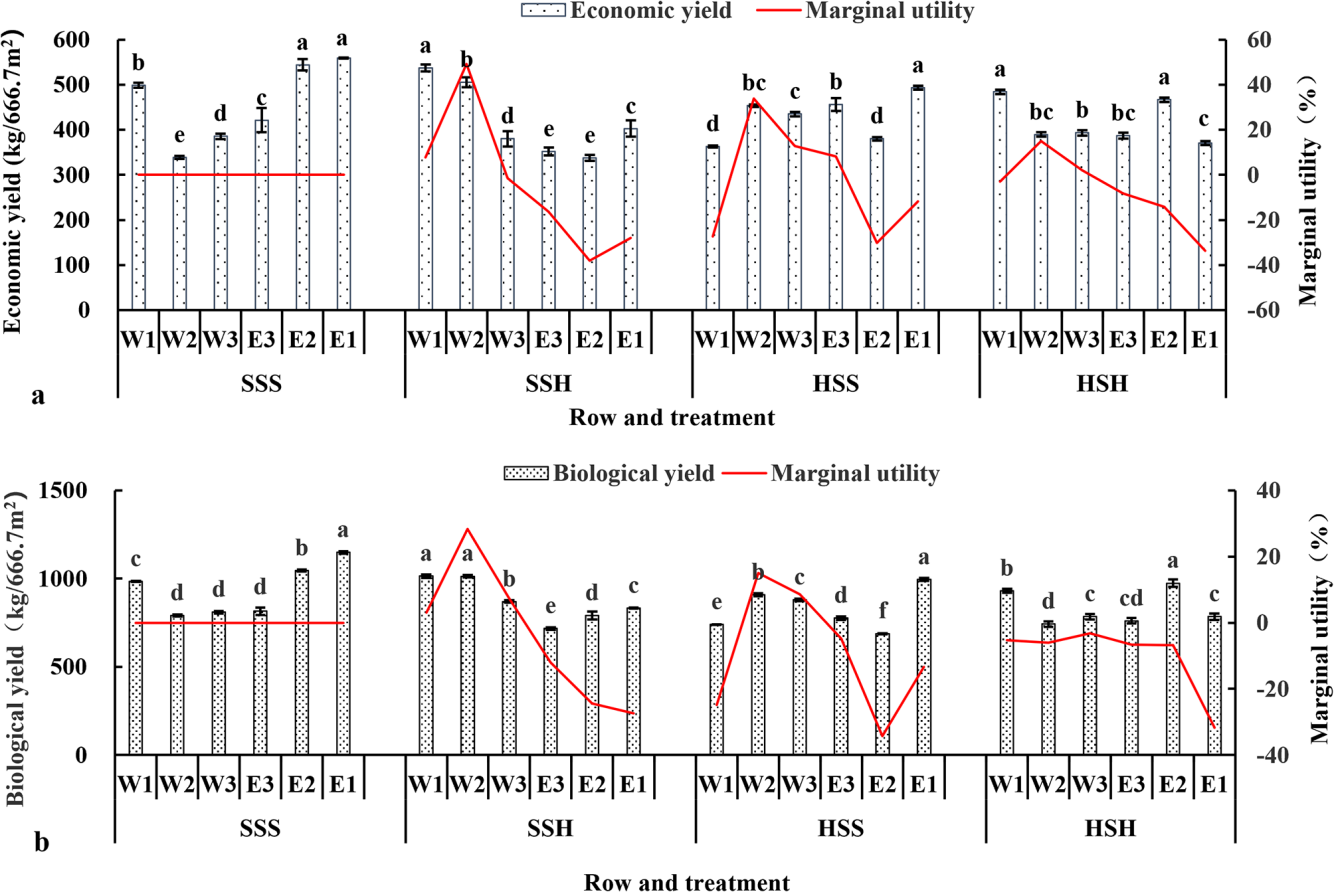
Changes in the economic and biological yields of the short-stem wheat varieties in each row of the experimental plot. (**a**: changes in economic yield, **b**: changes in biological yield)

Figure 6(a and b) shows that the economic and biological yields of each row showed a consistent change trend, and the economic and biological yields of W1 and E1 under SSS were significantly greater than those of W2, W3 and E3, indicating the significant positive marginal utility of the side row. However, the economic and biological production of E1 in the SSH, W1 in the HSS and E1 in the HSH was lower than that in the SSS, and the marginal utility was negative compared to that in the SSS. Except for W2 and W3 under HSH, the marginal utility of economic yield in the other rows was negative compared with that under SSS, while the marginal utility of biological yield in all rows under HSH was negative.

**Fig. 7:**
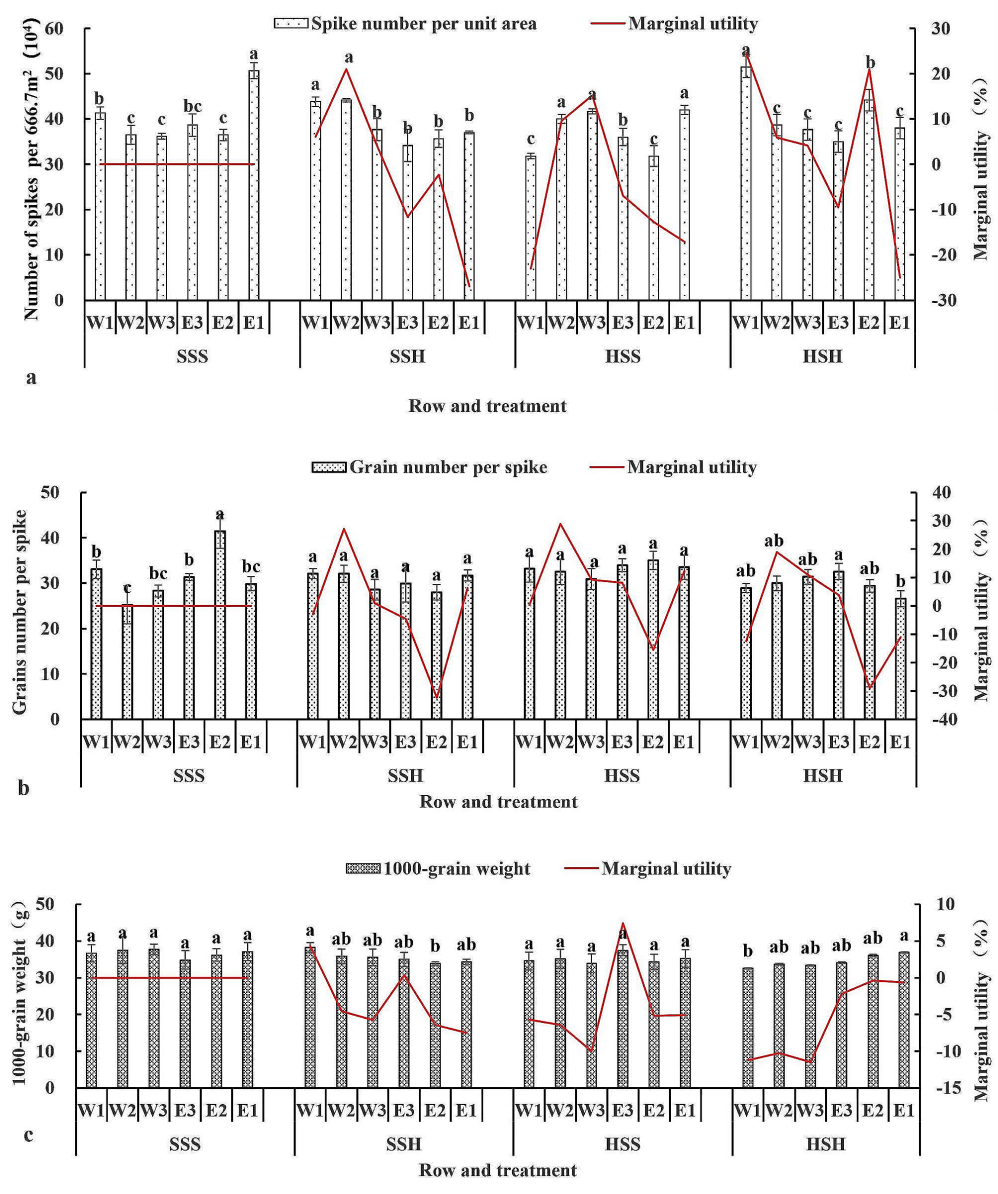
Changes in the three economic yield factors of each row in the experimental plot of the short-stem wheat varieties. (**a**: changes in spike number per unit area, **b**: changes in grain number per spike, **c**: changes in 1000-grain weight)

Figure 7(a) shows that, compared with those of SSS, the marginal effects of the spike number per unit area of E1, E2, and E3 in SSH were all negative, while the marginal effects of the spike number per unit area of W1, E3, E2, and E1 in HSS were all negative. The marginal effects of the spike number per unit area in E1 and E3 under HSH were negative, while the marginal effects of the spike number per unit area in the other rows under HSH were positive. The SSH, HSS, and HSH treatments had negative effects on the spike number per unit area in 9 rows, while the average marginal effect on the spike number per unit area in the experimental treatment plot was negative.

Figure 7(b) shows that, compared with those of SSS, the marginal effects of W1, E3, and E2 in SSH; E2 in HSS; and W1, E2, and E1 in HSH were negative, while those of the other rows were positive. The average marginal effect of the grain number per spike in the three experimental treatment plots was negative.

Figure 7(c) shows that, compared with those of SSS, the marginal effects of W1, E3, and E2 on the 1000-grain weight in SSH were positive, while those in the other rows were negative. The average marginal effects on the 1000-grain weight in the three experimental treatments were negative.

**Fig. 8:**
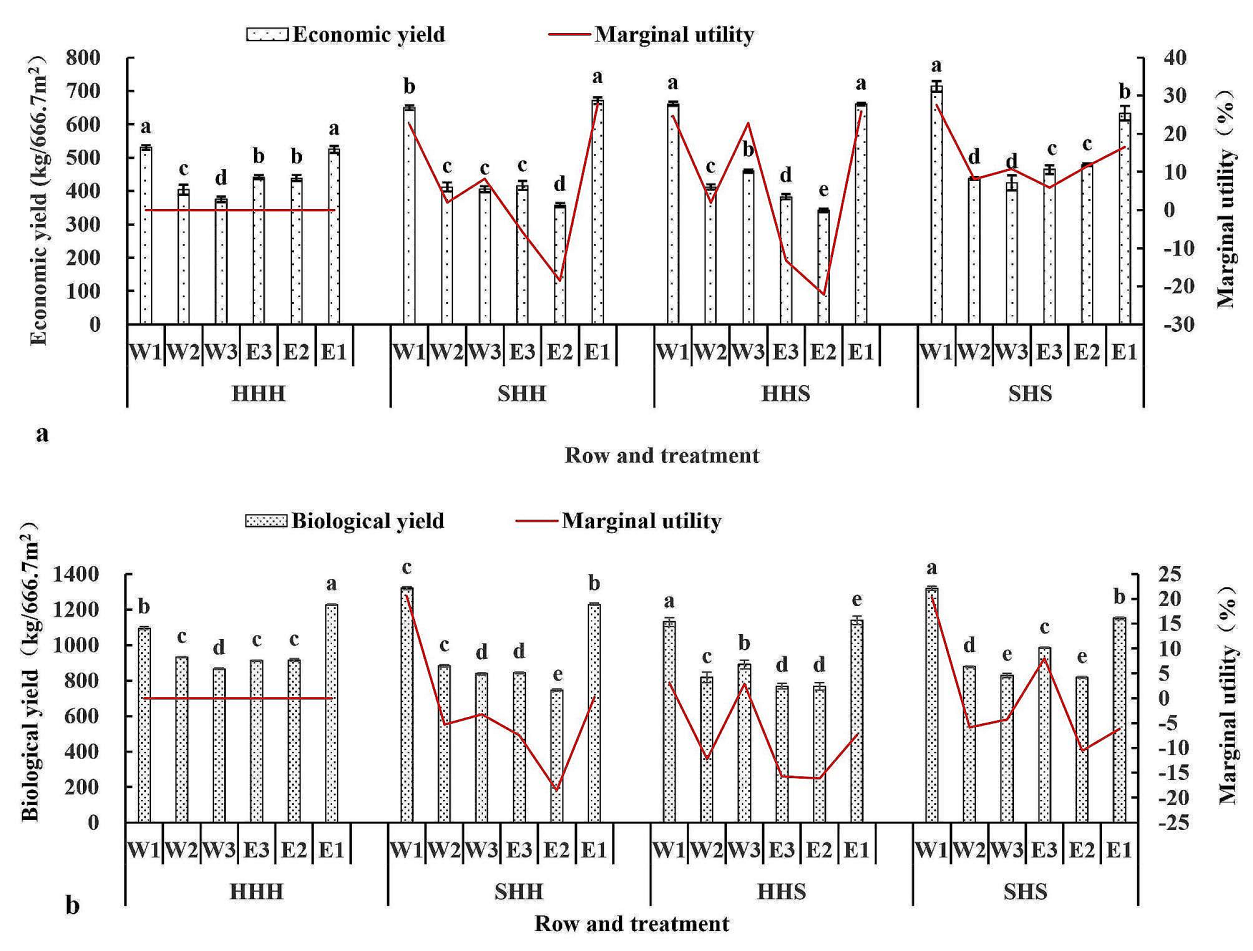
Changes in the economic and biological yields of the high-stem wheat varieties in each row of the experimental plot. (**a**: changes in economic yield, **b**: changes in biological yield)

Figure 8(a and b) shows that the biological and economic yields of W1 and E1 in each treatment were significantly greater than those in the other rows. Compared with those of HHH, the marginal effects of the economic yield of E3 and E2 in SHH and HHS were negative, while the marginal effects of the economic yield in the other rows were positive. The marginal effects of the economic yield in all rows of SHS were positive. Compared with those in the HHH treatment, the marginal effects of W1 and E1 in the SHH treatment, of W1 and W3 in the HHS treatment, and of W1 and E3 in the SHS treatment on biological production were positive, while the other rows had negative effects. The average marginal effect of economic production in each row of the three treatments was positive, while the average marginal effect of biological production was negative.

**Fig. 9:**
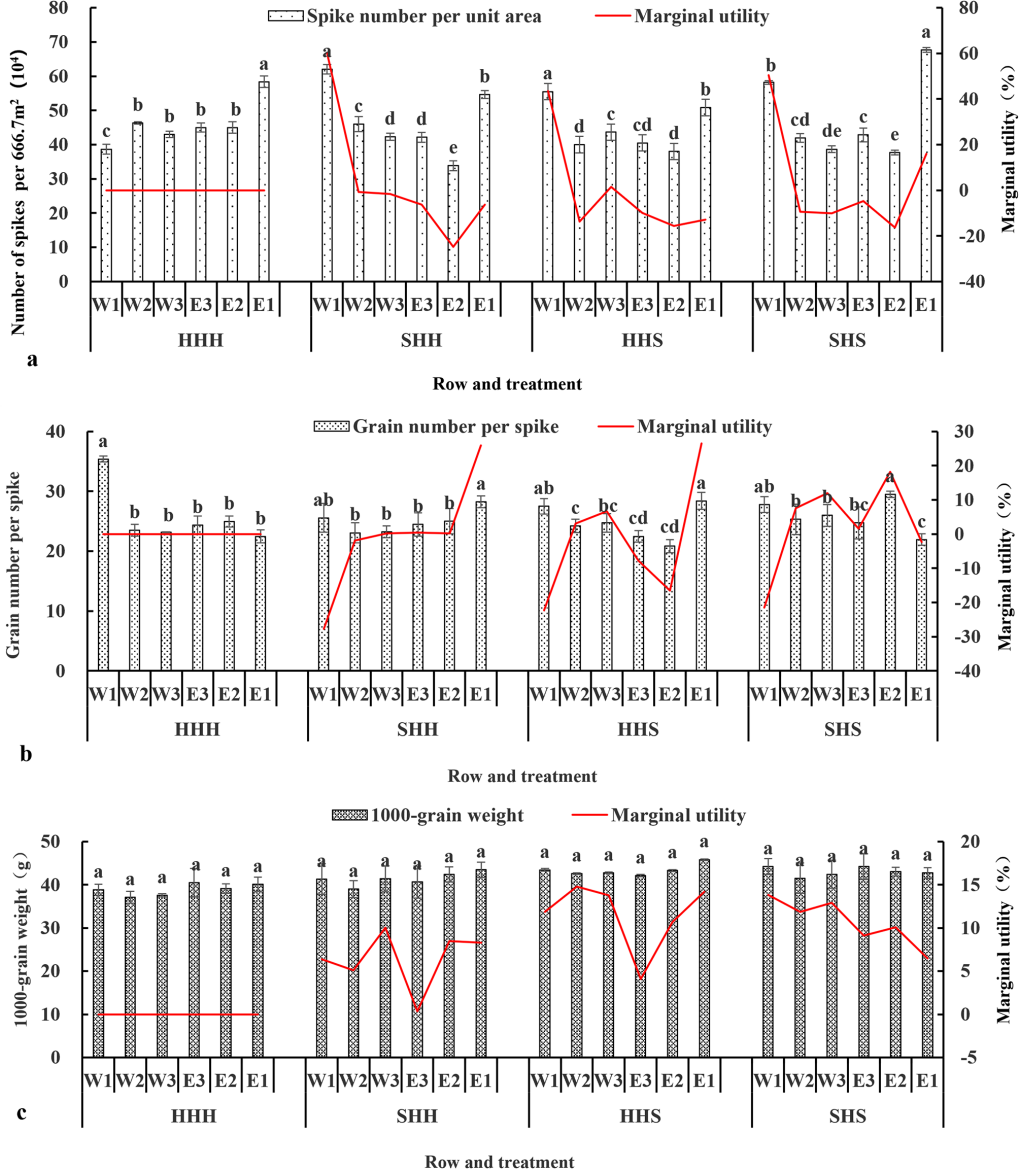
Changes in the three economic yield factors of each row in the experimental plot of the high-stem wheat varieties. (**a**: changes in spike number per unit area, **b**: changes in grain number per spike, **c**: changes in 1000-grain weight)

As shown in Fig. 9(a, b, and c), the three yield factors in each treatment row exhibited different patterns of change. Compared with those of HHH, the marginal effects of W1 in SHH, W1 and W3 in HHS, and W1 and E1 in SHS were all positive, while the marginal effects of the number of spikes per unit area in the other rows were negative. Compared with those of HHH, the marginal effects of the grain number per spike of W1 and W2 in SHH; of W1, E3, and E2 in HHS; and of W1 and E1 in SHS were negative, while the marginal effects of the grain number per spike in the other rows were positive. Compared with those of HHH, the marginal effects of the 1000-grain weight on SHH, HHS, and SHS were all positive. The average marginal effects of the number of spikes per unit area, number of grains per spike, and 1000-grain weight in the three experimental treatments were all positive.

### Changes in the economic yield and marginal effects of adjacent high-stem and short-stem wheat varieties

**Fig. 10:**
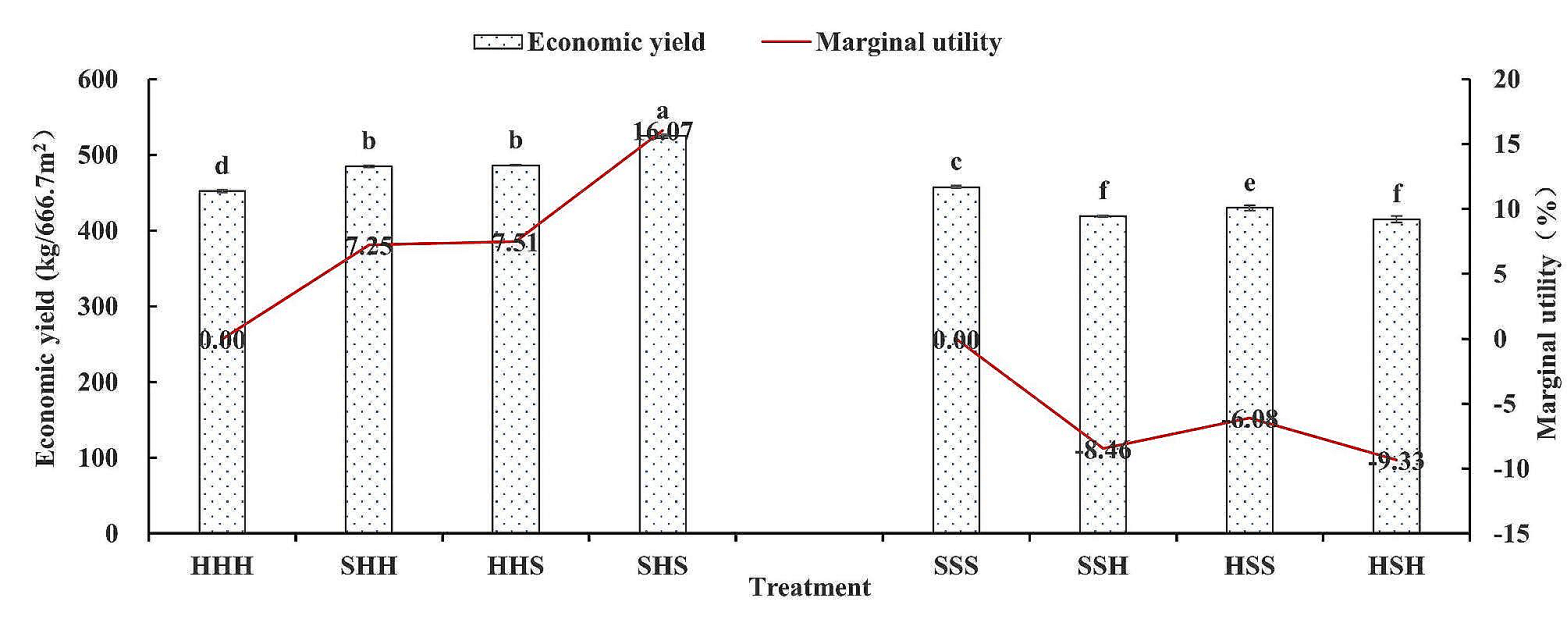
Economic yield changes in different plots of adjacent high-stem and short-stem wheat varieties

Figure 10 shows that the economic yields of SHH, HHS, and SHS were significantly greater than those of HHH, with marginal effects of economic yields of 7.25%, 7.51%, and 16.07%, respectively, and an average increase of 10.28%. However, the economic yields of SSH, HSS, and HSH were significantly lower than those of SSS, with marginal effects of economic yields of -8.46%, -6.65%, and − 9.33%, respectively, and an average economic yield reduction of 7.96%. The economic yield of the SSS plots was significantly greater than that of the HHH plots, and the economic yields of the SHH, HHS, and SHS experimental plots were significantly greater than those of the SSS, SSH, HSS, and HSH plots.

## Conclusion

In the regional wheat experiment, when high-stem and short-stem wheat varieties were planted adjacent to each other, the difference in plant height led to changes in soil temperature, soil moisture, and canopy illumination in the experimental plot, which affected the chlorophyll fluorescence index of the wheat flag leaves, resulting in changes in economic yield, biological yield, and three yield factors in each treatment compared to those in the control.

The final finding was that the marginal effect of economic yield was positive for the high-stem wheat varieties, while the marginal effect was negative for the short-stem wheat varieties. This study revealed that the average yield increase in the Xinhuamai818 SHH, HHS, and HSH treatments was 10.28%, while the average yield decrease in the Bainong307 SSH, HSS, and HSH treatments was 7.96%. Therefore, planting high-stem wheat varieties adjacent to short-stem wheat varieties in experimental plots may lead to the inability to objectively evaluate the economic yield of both varieties.

### Discussion and suggestions

In crop production experiments, marginal utility is an objective ecological phenomenon. When short-stem crops are planted adjacent to high-stem crops, short-stem crops have a positive effect on yield of the high-stem crops, while high-stem crops have a negative effect on yield of the short-stem crops [8–12]. Research by Li Xuejun, Ou Xingqi, and others has shown that different wheat varieties have different edge row advantages. Hybrid progeny should be reasonably selected for wheat breeding, after which the impact of edge row advantages on plot economic yield should be determined via plot experiments [26–28]. Li Xuejun’s research suggested that marginal effects in regional wheat trials directly affect the economic yield and rank of various wheat materials [26]. When the height of crops in adjacent plots is different, the marginal effect is particularly significant [15, 20, 25]. Our research revealed not only the existence of edge effects in crop experiments but also the mechanism and degree of influence of high-stem wheat plots on short-stem plots.

In the wheat planting area in the Huang-Huai-Hai region of China (located in the Northern Hemisphere), when wheat is planted in a north‒south direction (i.e., the plots are arranged east‒west), the marginal utility of the small plots is particularly evident. Due to the variation in the solar radiation angle throughout the day, small plots of high-stem wheat varieties will provide shade to small plots of short-stem wheat varieties, especially in April and May, and the heights of high-stem and short-stem wheat varieties will be fixed. This shading not only directly affected the lighting conditions of the short-stem wheat variety plot but also indirectly affected the changes in soil temperature, humidity, and chlorophyll fluorescence ability of the flag leaves and thus significantly reduced the economic yield and yield factors of the short-stem wheat varieties compared to those of the control group. These impacts will ultimately lead to biases in the evaluation of the economic yield of the plot.

The purpose of plant breeding is to select varieties with higher yields than the control [29], while regional variety trials are used to officially identify whether the economic yield of a variety is greater than that of the control. In regional wheat trials, objective evaluation of crop yield is very important because it directly determines whether a variety is further involved in production experiments and variety approval. If crop yield cannot be objectively evaluated in a variety of regional trials, this may affect the selection and promotion of new varieties. In 2021, the China Crop Variety Approval Committee released the national level rice and corn variety approval standards (revised in 2021), which state that for special types of varieties, applicants can propose variety approval standards based on actual production needs, submit them to the National Crop Variety Approval Committee for approval, and conduct variety experiments on their own. Regional trials of new wheat varieties can be found according to the approval standards for new rice and corn varieties, and separate experimental plots can be arranged for regional trials of special types of varieties, especially short-stem wheat varieties, to objectively evaluate the economic yield of every wheat variety.

In recent years, the approval of new wheat varieties in China has gradually improved, providing larger experimental plots for some special types, especially short-stem wheat varieties, to reduce the impact of marginal effects on yield. In addition, according to the objective phenomenon of marginal effects in agricultural production, the height of adjacent crops should be fully considered in crop production to avoid excessive marginal effects that affect crop yield and quality. How to scientifically and reasonably reduce the impact of edge row advantage effects on yield in regional wheat trials is a topic worthy of in-depth exploration.

In regional wheat trials, when high-stem and short-stem wheat varieties are planted adjacent to each other in small plots, the yield of high-stem wheat varieties significantly increases, while the yield of short-stem wheat varieties significantly decreases, making it difficult to objectively evaluate the yield performance of wheat varieties. To ensure the accuracy and impartiality of the experimental results, the following options can be considered in regional wheat trials: setting up independent experimental areas for short-stem wheat varieties during regional wheat trials or arranging three short-stem wheat plots consecutively and using the middle plot as the basis for yield evaluation. When arranging regional experiments, adjacent planting of wheat varieties with similar plant heights can be arranged; alternatively, increasing the number of rows in a single plot can reduce the impact of marginal effects (as the greater the number of rows in the plot is, the lower the proportion of side rows), or increasing the width of the plot ridge can reduce the marginal utility of adjacent plots.

## Data Availability

The datasets used and/or analysed during the current study are available from the corresponding author on reasonable request.
